# Effect of stroke direction on plantar pressure in each foot during the forehand and backhand stroke among healthy adult tennis players of different performance levels

**DOI:** 10.1186/s13102-023-00632-4

**Published:** 2023-02-23

**Authors:** Johanna Lambrich, Thomas Muehlbauer

**Affiliations:** Division of Movement and Training Sciences/Biomechanics of Sport, University of Duisburg-Essen, Gladbecker Str. 182, 45141 Essen, Germany

**Keywords:** Racket sport, Lower extremity, Pressure-detecting insoles, Plantar loading, Force, Biomechanics

## Abstract

**Background:**

In tennis, previous studies have shown differences in plantar pressure depending on tennis-specific movements (i.e., baseline play, serve & volley play, change of direction), playing surface (e.g., hard, grass, or clay), and serve type (e.g., slice, topspin or flat). However, the influence of stroke direction on plantar pressure in tennis players with diverging skill level is unknown. Thus, the purpose of this study was to determine the effect of stroke direction on plantar pressure in each foot during the forehand and backhand stroke among players of different performance levels.

**Methods:**

Thirty-nine female and male healthy adult tennis players (mean ± SD age: 23.5 ± 6.4 years) representing athletes from three performance levels (recreational, intermediate, advanced) participated in this study. The players performed longline/cross forehand and backhand groundstrokes (topspin) on a clay court while plantar pressure distribution was measured in each foot using flexible instrumented insoles.

**Results:**

The three-way ANOVA (performance level × stroke direction × foot dominance) showed (a) no significant differences in plantar pressure data between cross and longline strokes in almost all cases, (b) in part, significantly larger pressure values in advanced compared to intermediate and recreational players, and (c) significantly larger pressure data for the dominant compared to the non-dominant foot in nearly all comparisons.

**Conclusion:**

Regarding an appropriate plantar pressure distribution, our results suggest that during training of especially recreational and intermediate players attention should be paid to the feet rather than to stroke direction.

## Background

In tennis, stroke direction (i.e., cross or longline) in combination with stroke technique (i.e., forehand or backhand) and stroke type (i.e., slice or topspin) is a significant factor for variable groundstrokes during a match [1]. Accordingly, some studies [2, 3] investigated the influence of different characteristics of the previously mentioned stroke factors on biomechanical variables during tennis-specific movements. Specifically, plantar pressure data were analysed and the results showed higher values for the flat versus the twist serve and for the foot-up versus the foot-back stance style in competitive tennis players (*N* = 10, mean ± SD age: 23.8 ± 6.0 years) [2]. In addition, junior tennis players (*N* = 15, age range: 10–16 years, tournament level) showed higher plantar pressure values for the flat compared to the topspin and the slice serve [3]. However, the aforementioned differences were not uniform, but differed between the feet (front foot and back foot), indicating different functional involvement (e.g., force production or stabilizing function) [3, 4].

Although the previously reported studies have increased the knowledge on the influence of different characteristics of tennis serve and foot placement on plantar pressure distribution, studies on the influence of stroke direction (i.e., cross and longline) are lacking. In addition, all previous studies only examined players representing a single performance level and based on studies on physical [5, 6] and biomechanical [7, 8] variables in tennis, it is reasonable to assume that plantar pressure distribution differs as a function of performance level. Indeed, higher plantar pressure during stroke has been reported for elite compared to sub-elite table tennis [9] and badminton players [10]. However, such comparisons have so far not been carried out for tennis players and transferring the aforementioned findings to tennis is questionable because these types of sports have different underlying physical, technical, and tactical requirements [5, 11, 12].

Therefore, the aim of the present study was to investigate the effect of stroke direction (i.e., cross vs. longline) on plantar pressure distribution in each foot (i.e., dominant vs. non-dominant) during the forehand and backhand groundstroke among healthy adult tennis players of different performance levels (i.e., recreational, intermediate, or advanced). We hypothesised that plantar pressure data will differ between cross and longline stroke direction as well as among dominant and non-dominant foot, and this will further be affected by players’ performance level. To the best of our knowledge this is the first study that investigated the effect of stroke direction (i.e., cross vs. longline) on plantar pressure per foot during the forehand and backhand stroke in tennis players of different performance levels. From a practical point of view, the present study has the potential to influence the design of training programs for performance enhancement. Precisely, with a better understanding of plantar pressure differences in terms of stroke direction and foot dominance depending on performance level, specifically tailored exercises can be designed.

## Methods

### Participants

Thirty-nine female and male healthy adult tennis players from different local tennis clubs agreed to participate in this study (Table 1). Players were assigned to one of three study groups depending on their performance level. Players with an International Tennis Number (ITN) 2–3 were assigned to the "advanced" group, while subjects with ITN 4–6 belonged to the "intermediate" group, and individuals with ITN 7–10 were classified as "recreational". Participants’ written informed consent were obtained prior to the start of the study. The human ethics committee at the University of Duisburg-Essen, Faculty of Educational Sciences approved the study protocol.

**Table 1:** Characteristics of the study participants (*N* = 39) by group

Characteristic	Advanced tennis players (*n* = 13)	Intermediate tennis players (*n* = 13)	Recreational tennis players (*n* = 13)
ITN	2–3	4–6	7–10
Sex [f/m]	7/6	7/6	8/5
Age [years]	25.9 ± 7.1	23.5 ± 7.1	21.1 ± 4.0
Body height [cm]	180.5 ± 6.3	173.4 ± 12.0	176.9 ± 11.8
Body mass [kg]	71.8 ± 7.0	69.7 ± 12.7	69.9 ± 11.5
Training experience [years]	19.7 ± 6.3	16.2 ± 6.4	11.6 ± 7.0
Training volume [hours/week]	4.1 ± 1.8	3.7 ± 2.7	1.5 ± 1.1

Data represent means ± standard deviations

*ITN* International tennis number; *f* Female; *m* Male

### Testing procedure

**Fig. 1:**
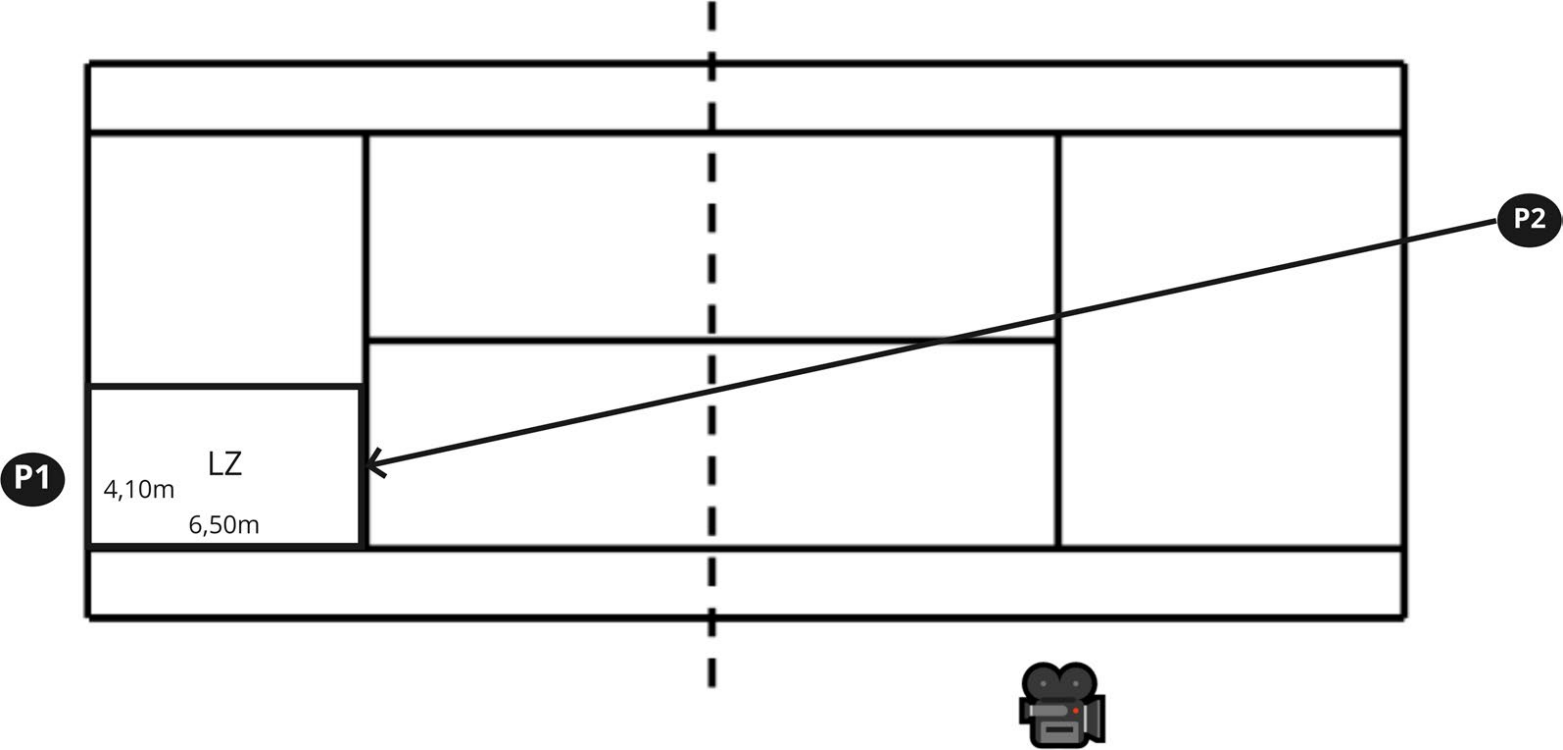
Experimental setup for the forehand and backhand cross strokes. P1 = player 1 (i.e., feed); P2 = player 2 (i.e., return); LZ = Landing zone

During a single visit to the clay court, tennis players performed a standardized 15-min tennis-specific warm-up including ground strokes with submaximal speed. Afterwards, the players were familiarized with the instrumented insoles followed by the execution of different groundstrokes (topspin) using a standardized sequence: a) forehand cross, b) backhand cross, c) forehand longline, and d) backhand longline. The players were free to decide their stance condition (i.e., open, closed, square). A trial included the feed at submaximal speed by player 1, followed by the return stroke at submaximal speed by the investigated player 2 into a predetermined 4.12 × 6.50 m landing zone (Fig. 1). Using this procedure, a rally was performed until six successful trials were completed per stroke technique. In order to avoid fatigue, a rest period of 120 s was provided before the next stroke technique was performed. Due to measurement errors (e.g., transmitter errors), the average of four successful trials per outcome variable was used for subsequent analysis.

### Assessment and analysis of plantar pressure data

**Fig. 2:**
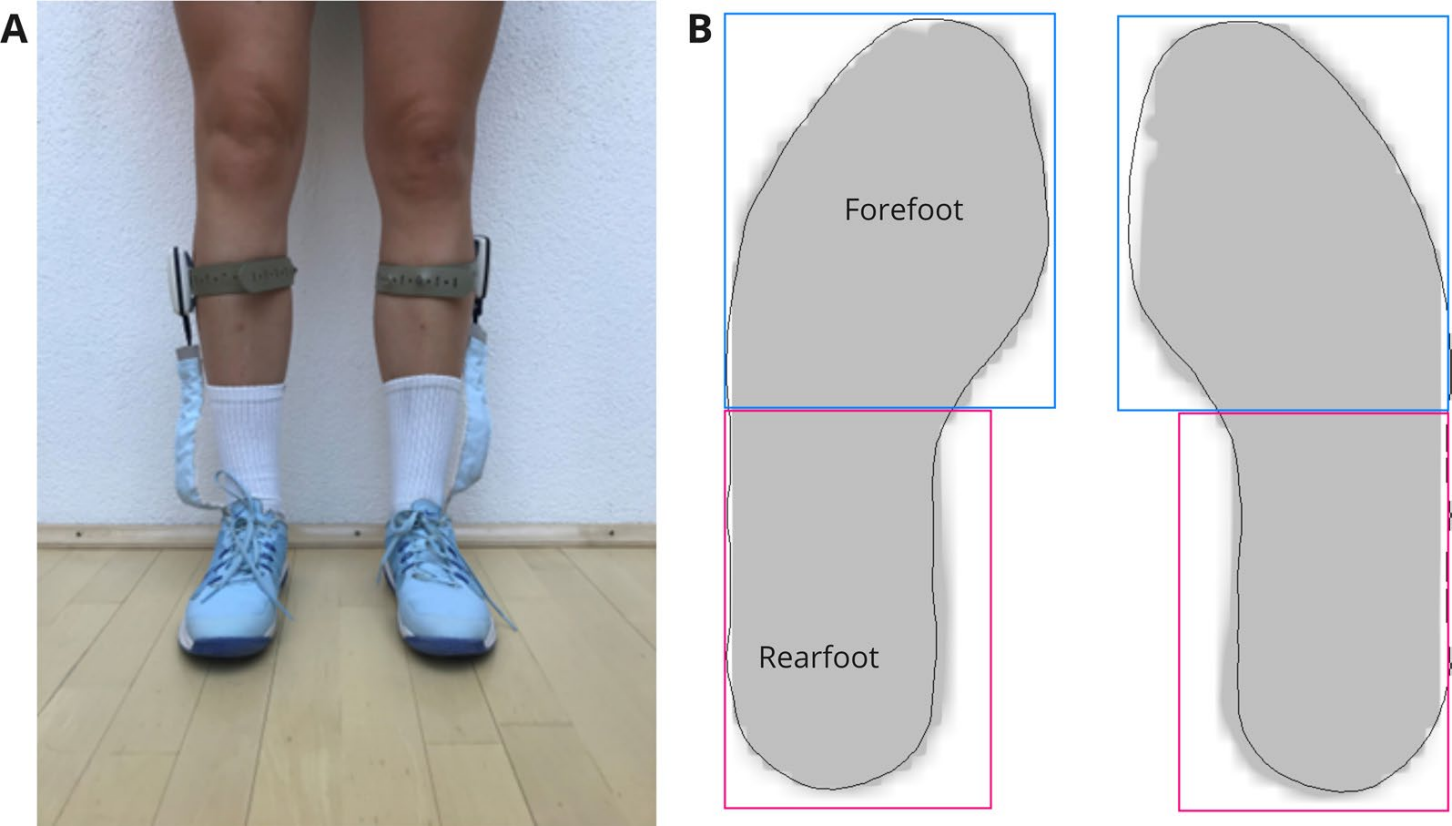
Illustration of a player wearing the mobile plantar pressure measurement system (**A**) and the used foot zone classification (**B**)

Plantar pressure distribution was recorded using flexible instrumented insoles (GP MobilData WiFi, GeBioM mbH, Münster, Germany) with a sampling frequency of 200 Hz (Fig. 2A). The obtained data were sent to a laptop via a wireless signal. Synchronously to the pressure data, players’ movement was filmed using a video camera (Fig. 1). Precisely, prior to each condition, one standardized movement was performed per player, which was clearly identifiable in both the video and the pressure-detecting insoles. Subsequently, the start of a stroke was defined as the beginning of the forward swing and the end of a stroke as the completion of the follow through phase. Due to variable durations of stroke execution, the data were normalised and interpolated from 0 to 100% of the stroke cycle. Two-hundred one data points were used for the interpolation. The analysis of the pressure data was performed for the whole foot and for the forefoot and rearfoot, separately (Fig. 2B) using Matlab software version R2017a (The MathWorks Inc., Natick, MA, USA). The exported force data represents the force “that exerts the same mechanical effect as the sum of the forces transmitted across the contact surface [13]”. Afterwards, the maximal force values were normalized to the players’ body mass (N/kg) and used for statistical analyses. Further, the force–time integral (Nms/kg) was calculated based on the normalized force data.

### Statistical analysis

All statistical analyses were performed with SPSS version 27.0 (IBM Corporation, Armok, NY, USA). Descriptive data are reported as group mean values and standard deviations. For all analyses, assumptions of normality (Shapiro–Wilk Test) and homogeneity of variance/sphericity (Mauchly Test) were checked and met prior to the application of analysis of variance (ANOVA). A 3 (performance level: recreational, intermediate, advanced) × 2 (stroke direction: cross, longline) × 2 (foot dominance: dominant (equals the stroke arm), non-dominant) repeated measures ANOVA was conducted for the forehand and the backhand, separately. If a significant performance level by stroke direction interaction occurred, Bonferroni-adjusted post-hoc tests (i.e., paired *t*-tests) were performed. Further, effect size (*η*_p_^2^) was calculated and reported as small (0.02 ≤ *η*_p_^2^ ≤ 0.12), medium (0.13 ≤ *η*_p_^2^ ≤ 0.25), and large (*η*_p_^2^ ≥ 0.26). The significance level was set at *p* < 0.05.

## Results

Descriptive statistics of the plantar pressure data for the whole foot, the forefoot, and rearfoot by stroke direction (i.e., cross vs. longline), performance level (recreational, intermediate, or advanced), and foot dominance (i.e., dominant vs. non-dominant) during the forehand and backhand groundstroke are illustrated in Table 2.

**Table 2:** Descriptive statistics of the plantar pressure data for the whole foot, the forefoot, and rearfoot by stroke direction (i.e., cross vs. longline), performance level (recreational, intermediate, or advanced), and foot dominance (i.e., dominant vs. non-dominant) during the forehand and backhand groundstroke (topspin)

Outcome	Cross						Longline					
	RP		IP		AP		RP		IP		AP	
	D	ND	D	ND	D	ND	D	ND	D	ND	D	ND
*Forehand stroke*												
Maximal force [N/kg]												
Whole foot	.846 ± .250	.704 ± .315	.966 ± .372	.829 ± .376	1.203 ± .408	.961 ± .400	.843 ± .337	.685 ± .293	1.046 ± .384	.805 ± .346	1.222 ± .331	.990 ± .508
Forefoot	.605 ± .240	.536 ± .338	.755 ± .387	.632 ± .393	.982 ± .413	.775 ± .341	.557 ± .233	.517 ± .317	.770 ± .380	.595 ± .352	.971 ± .312	.826 ± .501
Rearfoot	.391 ± .197	.307 ± .172	.363 ± .156	.286 ± .106	.336 ± .150	.308 ± .155	.395 ± .222	.265 ± .143	.442 ± .222	.312 ± .083	.345 ± .200	.284 ± .147
Force–time integral [Nms/kg]												
Whole foot	56.6 ± 23.2	53.3 ± 27.4	69.1 ± 26.2	60.5 ± 37.6	89.8 ± 40.7	86.3 ± 29.6	59.1 ± 29.1	49.6 ± 20.0	81.2 ± 38.6	44.5 ± 21.1	61.2 ± 26.0	64.2 ± 38.5
Forefoot	38.0 ± 17.4	34.1 ± 19.8	52.4 ± 25.9	45.6 ± 34.4	73.1 ± 37.8	48.3 ± 23.4	36.6 ± 20.1	34.1 ± 19.9	59.8 ± 34.7	30.3 ± 19.3	70.4 ± 28.9	52.8 ± 36.5
Rearfoot	18.3 ± 10.5	18.9 ± 14.3	16.4 ± 9.1	14.6 ± 8.53	16.5 ± 11.5	15.7 ± 13.6	22.2 ± 16.0	15.3 ± 9.7	21.1 ± 13.9	14.0 ± 7.2	15.7 ± 13.6	12.7 ± 8.8
*Backhand stroke*												
Maximal force [N/kg]												
Whole foot	.880 ± .261	.683 ± .282	.920 ± .315	.725 ± .269	1.114 ± .332	1.003 ± .459	.755 ± .211	.711 ± .323	.931 ± .329	.783 ± .368	1.039 ± .332	.945 ± .435
Forefoot	.580 ± .244	.487 ± .326	.681 ± .256	.486 ± .284	.891 ± .290	.818 ± .471	.570 ± .227	.510 ± .331	.699 ± .350	.612 ± .393	.794 ± .309	.811 ± .430
Rearfoot	.429 ± .252	.344 ± .117	.356 ± .197	.319 ± .122	.405 ± .143	.317 ± .151	.288 ± .186	.298 ± .151	.337 ± .126	.247 ± .132	.398 ± .163	.274 ± .132
Force–time integral [Nms/kg]												
Whole foot	72.8 ± 20.2	39.6 ± 23.9	82.9 ± 41.4	43.2 ± 31.9	90.7 ± 34.6	57.3 ± 26.6	60.9 ± 24.8	46.4 ± 28.5	83.1 ± 28.0	40.2 ± 20.2	57.3 ± 26.6	60.8 ± 44.2
Forefoot	47.7 ± 19.5	27.3 ± 21.7	57.6 ± 33.3	28.8 ± 27.7	69.2 ± 27.9	44.9 ± 30.1	43.1 ± 23.1	32.1 ± 23.7	58.8 ± 26.9	28.7 ± 19.7	59.3 ± 26.9	50.4 ± 40.9
Rearfoot	24.8 ± 15.5	12.1 ± 7.3	25.0 ± 18.2	14.1 ± 10.2	21.2 ± 12.2	12.2 ± 7.4	17.6 ± 15.1	14.1 ± 12.0	24.0 ± 14.7	11.4 ± 10.5	24.4 ± 12.8	10.3 ± 5.8

Data represent means ± standard deviations

*AP* Advanced players; *D* Dominant foot; *IP* Intermediate players; *ND* Non-dominant foot; *RP* Recreational players

### Forehand stroke

For the maximal force, the three-way ANOVA revealed significant main effects of performance level (except for the rearfoot; *p* = 0.030–0.042, *η*_p_^2^ = 0.16–0.18) and foot dominance (*p* < 0.001–0.008, *η*_p_^2^ = 0.18–0.33) but not of Stroke direction (Table 3). Further, there were no significant interaction effects. In terms of the force–time integral, the analysis yielded significant main effects of performance level (only for the forefoot: *p* = 0.019, *η*_p_^2^ = 0.20) and foot dominance (*p* < 0.001–0.028, *η*_p_^2^ = 0.13–0.35) but not of stroke direction (Table 3). Again, the interaction effects did not reach the level of significance. The main effects of performance level and foot dominance indicate that the plantar pressure data were greater a) in advanced compared to intermediate and recreational players and b) for the dominant compared to the non-dominant foot.

**Table 3:** Inference statistics for the main and interaction effects

Outcome	Main effect: SD	Main effect: PL	Main effect: FD	Interaction effect: SD × PL	Interaction effect: SD × FD	Interaction effect: PL × FD	Interaction effect: SD × PL × FD
*Forehand stroke*							
Maximal force [N/kg]							
Whole foot	.598 (.01)	.042 (.16)	< .001 (.33)	.787 (.01)	.534 (.01)	.736 (.02)	.704 (.02)
Forefoot	.734 (.01)	.030 (.18)	.008 (.18)	.671 (.02)	.812 (.01)	.511 (.04)	.672 (.02)
Rearfoot	.591 (.01)	.804 (.01)	< .001 (.27)	.163 (.10)	.308 (.03)	.491 (.04)	.981 (.01)
Force–time integral [Nms/kg]							
Whole foot	.565 (.01)	.100 (.12)	< .001 (.35)	.899 (.01)	.286 (.03)	.143 (.10)	.262 (.07)
Forefoot	.422 (.02)	.019 (.20)	< .001 (.30)	.435 (.05)	.517 (.01)	.108 (.12)	.219 (.08)
Rearfoot	.757 (.01)	.361 (.06)	.028 (.13)	.459 (.04)	.126 (.06)	.951 (.01)	.620 (.03)
*Backhand stroke*							
Maximal force [N/kg]							
Whole foot	.386 (.02)	.051 (.15)	.009 (.17)	.365 (.05)	.092 (.08)	.828 (.01)	.378 (.05)
Forefoot	.753 (.01)	.029 (.18)	.118 (.07)	.191 (.09)	.054 (.10)	.664 (.02)	.709 (.02)
Rearfoot	.014 (.16)	.747 (.02)	.003 (.22)	.404 (.05)	.957 (.01)	.420 (.05)	.273 (.07)
Force–time integral [Nms/kg]							
Whole foot	.220 (.04)	.096 (.12)	< .001 (.42)	.945 (.01)	.229 (.04)	.482 (.04)	.449 (.04)
Forefoot	.735 (.01)	.068 (.14)	< .001 (.32)	.758 (.02)	.458 (.04)	.197 (.05)	.516 (.04)
Rearfoot	.288 (.03)	.833 (.01)	< .001 (.33)	.508 (.04)	.797 (.01)	.795 (.01)	.143 (.10)

Values are expressed as *p*-value (*η*_*p*_^2^-value)

*FD* Foot dominance; *PL* Performance; *SD* Stroke direction

### Backhand stroke

With respect to the maximal force, the three-way ANOVA showed significant main effects of stroke direction (only for the rearfoot: *p* = 0.014, *η*_p_^2^ = 0.16), performance level (only for the forefoot: *p* = 0.029, *η*_p_^2^ = 0.18), and foot dominance (except for the forefoot; *p* = 0.003–0.009, *η*_p_^2^ = 0.17–0.22) (Table 3). Yet, there were no significant interaction effects. Considering the force–time integral, the analysis detected a significant main effect of foot dominance (all *p* < 0.001, *η*_p_^2^ = 0.32–0.42) but not of stroke direction and performance level (Table 3). In addition, none of the interaction effects reached the level of significance. The main effects of stroke direction, performance level and foot dominance indicate that the plantar pressure data were greater a) during cross compared to longline stroke, b) in advanced compared to intermediate, and recreational players, and c) for the dominant compared to the non-dominant foot.

## Discussion

The aim of this study was to investigate effects of stroke direction on plantar pressure in each foot during the forehand and backhand groundstroke (topspin) among healthy adult tennis players of different performance levels. The main results can be summarized as follows: (a) in all cases (except for the rearfoot during backhand stroke), there were no significant differences in plantar pressure data between cross and longline strokes; (b) in part, significantly larger pressure values were found in advanced compared to intermediate and recreational players as well as in intermediate compared to recreational players; (c) nearly all comparisons showed significantly larger pressure data for the dominant compared to the non-dominant foot.

### Plantar pressure by stroke direction

Contrary to our hypothesis, there were almost no differences in plantar pressure data between the cross and longline stroke (except for the maximal force of the rearfoot during the backhand stroke), regardless of the foot dominance and performance level considered. Accordingly, in contrast to other factors (e.g., serve type) stroke direction does not seem to have a significant influence on plantar pressure distribution. Alternatively, it could be argued that the assessment of plantar pressure data is not sensitive enough to detect existing differences. However this seems unlikely, as several studies have shown pressure differences using insoles with respect to types of tennis serve [3, 14], service stance styles [2], and foot dominance [4]. From a practical perspective, the lacking influence of stroke direction on plantar pressure distribution means that little attention has to be paid to a different pressure configuration between cross and longline stroke during training.

### Plantar pressure by performance level

In accordance with the hypothesis, partial differences in plantar pressure data were shown regardless of the stroke direction and foot dominance considered. Specifically, the largest pressure values (i.e., maximal force and force–time integral) were observed in the advanced players followed by the intermediate players and then the recreational players. The available literature does not indicate research related to plantar pressure data in tennis players of different performance levels. Therefore, we compared the results of our research to other types of sport (i.e., table tennis and badminton). Qian et al. [9] examined lower limb kinematics and plantar pressure during table tennis forehand loop in superior (*N* = 13, mean ± SD age: 20.1 ± 0.9 years) versus intermediate (*N* = 13, mean ± SD age: 21.2 ± 1.6 years) players. Besides others, they observed significantly larger pressure data (e.g., contact area of the midfoot and rearfoot) at backward-end and forward-end in superior compared to intermediate players. Further, Zhao and Li [10] investigated lower limb kinematics and plantar pressure in the backcourt forehand clear stroke between professional (*N* = 10, mean ± SD age: 23.7 ± 2.4 years) and amateur (*N* = 10, mean ± SD age: 22.5 ± 1.9 years) badminton players. Among others, significantly larger pressure values (e.g., pressure–time integral at the first metatarsal head region) were found in professional compared to amateur players. Referring to the present study, the presence of a greater force level could be a possible reason for the larger plantar pressure values in advanced compared to intermediate and recreational players. For example, in a recent systematic review with meta-analysis, Lambrich and Muehlbauer [6] demonstrated that elite versus sub-elite players have a higher level of lower-extremity muscle power and are thus able to generate higher plantar pressure values. In addition, they reported positive correlations between lower extremity muscle power and stroke performance (i.e., stroke velocity). Therefore, the production of high plantar pressure values also seems to be positively related to stroke performance, which should be investigated in future studies.

### Plantar pressure by foot dominance

Consistent with the hypothesis and other studies [3, 4], differences in plantar pressure data were mainly detected between the dominant and the non-dominant foot, regardless of stroke direction and performance level considered. Precisely, the pressure values (i.e., maximal force and force–time integral) were significantly larger for the dominant compared to the non-dominant foot (except for the maximal force of the forefoot during the backhand stroke). A possible reason for the larger pressure data in the dominant foot can be derived from tennis technique. For instance, for the majority of forehand strokes as well as during the backhand stroke from the closed stance, the dominant leg serves as the stem leg for power production [15]. Thus, it seems useful to consider the different types of stress on the dominant and non-dominant leg. While the dominant leg is exposed to a higher load in the basic strokes, this should be specifically taken into consideration during training.

### Limitations

There are some limitations in this study that need to be addressed. First, the feed at submaximal speed was not standardized (e.g., via ball machine) but performed by a player. This results in some variability in the fed ball speed, spin, and depth, which may have influenced the plantar pressure data. Second and due to the use of instrumented pressure-detecting insoles, the present results are limited to the vertical force component (F_Z_). Therefore, future investigations should be carried out using a force plate in order to be able to make statements on the other force components (i.e., F_Y_ and F_X_). Third, only pressure but no kinematic data of the lower extremities were collected. Therefore, no statements can be derived regarding changes in position data (e.g., angular displacements and velocities).

## Conclusions

We investigated the effect of stroke direction on plantar pressures in each foot during the forehand and backhand groundstroke (topspin) among healthy adult tennis players of different performance levels. Our findings suggest that out of the investigated parameters (a) stroke direction has almost no, (b) performance level has partial and (c) foot dominance has most influence on plantar pressure. Therefore, during the training of especially recreational and intermediate players, less attention should be paid to differences in plantar pressure between both stroke directions, but rather between the feet.

## Data Availability

The data generated and analysed during the present study are not publicly available due to ethical restrictions but are available from the corresponding author upon reasonable request.

## Abbreviations

AP: Advanced players
D: Dominant foot
f: Female
IP: Intermediate players
ITN: International tennis number
LZ: Landing zone
m: Male
ND: Non-dominant foot
P1: Player 1
P2: Player 2
RP: Recreational players
